# Selenium Accumulation Characteristics and Biofortification Potentiality in Turnip (*Brassica rapa* var. *rapa*) Supplied with Selenite or Selenate

**DOI:** 10.3389/fpls.2017.02207

**Published:** 2018-01-04

**Authors:** Xiong Li, Yuansheng Wu, Boqun Li, Yonghong Yang, Yongping Yang

**Affiliations:** ^1^Key Laboratory for Plant Diversity and Biogeography of East Asia, Kunming Institute of Botany, Chinese Academy of Sciences, Kunming, China; ^2^Germplasm Bank of Wild Species, Kunming Institute of Botany, Chinese Academy of Sciences, Kunming, China; ^3^Key Laboratory of Agro-Biodiversity and Pest Management of Education Ministry of China, Yunnan Agricultural University, Kunming, China; ^4^College of Plant Protection, Yunnan Agricultural University, Kunming, China

**Keywords:** selenium deficiency, turnip, selenite, selenate, tibetan plateau

## Abstract

Selenium (Se) is an essential trace element for humans. About 70% of the regions in China, including most of the Tibetan Plateau, are faced with Se deficiency problems. Turnip is mainly distributed around the Tibetan Plateau and is one of the few local crops. In the present study, we compared the absorption and translocation differences of Se (IV) selenite and Se (VI) selenate in turnip. The results showed that Se treatment, either by soil addition (0.2–2 mg Se kg^−1^ dry soil) or by foliar spraying (50–200 mg L^−1^ Se), could significantly increase the Se concentrations in turnips, and 0.5 mg Se (IV) or Se (VI) kg^−1^ dry matter in soils could improve the biomasses of turnips. Moreover, turnip absorbed significantly more Se (VI) than Se (IV) at the same concentration and also transferred much more Se (VI) from roots to leaves. Based on the Se concentrations, as well as the bioconcentration factors and translocation coefficients, we considered that turnip might be a potential Se indicator plant. Subsequently, we estimated the daily Se intake for adults based on the Se concentrations in turnip roots. The results indicated that Se (IV) should be more suitable as an artificial Se fertilizer for turnips, although the levels found in most samples in this study could cause selenosis to humans. In addition, we also estimated the optimum and maximum Se concentrations for treating turnips based on the linear relations between Se concentrations in turnip roots and Se treatment concentrations. The results provided preliminary and useful information about Se biofortification in turnips.

## Introduction

Selenium (Se) is an essential trace element for human beings and animals (White and Brown, 2010). Se deficiency is a serious threat to human health, and is associated with cardiovascular disease, a weakened immune system, hypothyroidism, male infertility, cognitive decline and increased risks of various cancers (Fairweather-Tait et al., 2011; Rayman, 2012; White, 2016). The World Health Organization (WHO) recommended a dietary allowance of about from 55 to 200 μg Se d^−1^ for different groups of people (Wu et al., 2015). Unfortunately, a great number of people around the world may lack sufficient Se for their well-being, mainly because of the uneven distribution of Se resources on the earth (Combs, 2001; Fairweather-Tait et al., 2011; Joy et al., 2014; Wu et al., 2015; White, 2016). Taking China as an example, approximately 70% of its regions are faced with Se deficiency in varying degrees (Zhu et al., 2009; Wu et al., 2015), though several areas are also found to possess seleniferous soils (Wu et al., 2015). Because much of Se in human bodies is derived either directly or indirectly from edible plants, Se in diet greatly depends on grain and vegetable production in soils with substantial Se content or Se phytoavailability (Broadley et al., 2006; White and Broadley, 2009; Chilimba et al., 2011; Joy et al., 2015). However, excessive dietary Se intakes can also produce toxic effects in humans and animals (Fairweather-Tait et al., 2011; Rayman, 2012; Sperotto et al., 2014). The symptoms of selenosis in humans are similar to those caused by heavy metals, and include dermatitis, cracking of nails, hair loss, garlicky breath, acute respiratory distress, myocardial infarction, and renal failure (White, 2016). The Institute of Medicine (USA) has suggested a tolerable upper intake of 400 μg Se d^−1^ for adults (White, 2016).

Although some studies have focused on the potential of plants with high-Se accumulation capacities in phytoremediation of Se-contaminated soils (Banuelos and Dhillon, 2011; Wu et al., 2015), much interest has been developed toward the value of crops, vegetables, and edible mushrooms (Zhao et al., 2004; Maseko et al., 2014; Dogan et al., 2016), for producing Se-enriched food because of the severe problem of Se deficiency. Indeed, the application of inorganic Se fertilizers, especially in non-seleniferous areas, has been an effective way to increase Se content of diets and to improve the Se status and health of both animals and humans (White and Broadley, 2009; Alfthan et al., 2015). Se concentrations in plants are directly associated with both Se phytoavailability in the soil and the ability of plants to accumulate Se (Dhillon and Dhillon, 2009). Angiosperm species can be divided into three ecological types based on their Se accumulation ability in tissues, which include non-accumulator, Se-indicator and Se-accumulator species (White et al., 2007; White, 2016). Most angiosperm species belong to non-accumulator species, which are unable to tolerate tissue Se concentrations >10–100 mg Se kg^−1^ dry weight (DW) and can hardly survive in seleniferous soils (Dhillon and Dhillon, 2009; White, 2016). In comparison, the Se-indicator species can tolerate tissue Se concentrations approaching 1 g Se kg^−1^ DW and survive in both non-seleniferous and seleniferous soils (Moreno Rodriguez et al., 2005; White, 2016). For Se-accumulator species, whose distribution is usually constrained within seleniferous soils, their tissue Se concentrations can exceed 1 g Se kg^−1^ DW (White, 2016). In particular, an extreme sub-set of Se-accumulator species are defined as Se-hyperaccumulators, with leaves containing at least 1 g Se kg^−1^ DW in natural environments (Terry et al., 2000), although some scientists suggested that this threshold should be lowered to 100 μg Se g^−1^ DW (van der Ent et al., 2012). To date, known Se-accumulator species include several members of Asteraceae, Brassicaceae, and Fabaceae, reported from America, Australia and China, respectively (Freeman et al., 2006, 2010; Yuan et al., 2013; White, 2016). These species accommodate high Se concentrations in leaf trichomes and epidermal cells (Freeman et al., 2006, 2010). Several members of the Lecythidaceae family are also well-known for accumulating high Se concentrations in their fruits and seeds (Chang et al., 1995; Hammel et al., 1996; Dernovics et al., 2007).

Brassicaceae species have always attracted much attention for their Se accumulation characteristics (Suarez et al., 2003; Seppanen et al., 2010; Hladun et al., 2011). The major reason is that Brassicaceae plants include a large proportion of the commonly cultivated vegetables around the world, such as pakchoi, cabbage, broccoli, mustard, radish and turnip, etc. Undoubtedly, Brassicaceae crops provide a considerable way to supplement Se intake for humans. However, Brassicaceae species, containing a large number of members (Couvreur et al., 2010), may have variable Se accumulation abilities that range from non-accumulator to Se-hyperaccumulator species (Suarez et al., 2003; Seppanen et al., 2010; Yuan et al., 2013). Therefore, it is necessary to independently discover the Se accumulation ability of each individual species. Turnip (*Brassica rapa* var. *rapa*), a cruciferous biennial plant, has been widely cultivated as a vegetable or fodder crop in Europe, America and Asia over a long history. It is rich in vitamin C, riboflavin, dietary fiber, and a variety of mineral elements, but is low in calories (Parveen et al., 2015). It is also considered to have antioxidants and can lower the risk of high blood pressure and diabetes, as well as various cancers (Parveen et al., 2015). In China, the distribution center of turnip is the Tibetan Plateau and its surrounding areas, which are faced with severe Se deficiency problems. Thus, it is of great interest to explore the absorption and accumulation characteristics of this species in China for Se nutrition supply to local people. Few relevant studies have been performed to date, according to a recent review from China (Wu et al., 2015).

In natural environments, selenite and selenate are the main water-soluble forms of Se in oxic and anaerobic soils, respectively (White, 2016). Plants usually show differential accumulation ability for these two Se forms and they may have different toxicity thresholds in plants (Fu et al., 2011; Mao et al., 2011; Longchamp et al., 2015; Schiavon et al., 2016). In the present study, we compared the absorption and translocation differences of selenite and selenate in turnip and estimated the Se intake safety in turnip foodstuffs based on the experimental results. We would like to understand the ability of turnips to accumulate Se and to assess their potential for producing Se-supplemented food in natural seleniferous soils or via artificial Se fertilizers.

## Materials and methods

### Plant cultivation and treatment

Turnip seeds were collected from Ninglang County of China, from a population constituting a local landrace (NO. KTRG-B54) (Li et al., 2016). The seeds were germinated and grown outdoors in mid-April. To compare the differences of absorption and translocation of Se forms of Se (IV) selenite and Se (VI) selenate in turnip, concentrations of 0, 0.2, 0.5, and 2 mg Se kg^−1^ DM were established through fertilization of artificial Se-free mucky soil (Table 1) with sodium selenite (Na_2_SeO_3_) and sodium selenate (Na2SeO4), respectively. Three uniform 35 × 28 × 21-cm boxes were prepared for each Se treatment and 5 kg mucky soil was fitted into the boxes. After 25-day growth, three turnip seedlings with consistent size were neatly transplanted in each box. The boxes were placed under a transparent plastic shed with appropriate watering. At 45 day post-transplantation, the plants of each treatment were harvested for subsequent measurement.

**Table 1:** Parameters of the soil used in the experiment.

**Parameter**	**Unit**	**Soil D**
pH	/	7.44
Organic matter	g kg^−1^ DW	332.9
Humus	g kg^−1^ DW	191.2
Total N	g kg^−1^ DW	0.79
Total P	g kg^−1^ DW	0.37
Available K	mg kg^−1^ DW	275.2
Exchangeable Ca	cmol kg^−1^ DW	12.15
Exchangeable Mg	cmol kg^−1^ DW	1.18
Total Se	mg kg^−1^ DW	ND^a^

a *ND indicates that the data were not detected. The detection limit in the experiment was 0.02 mg kg^−1^*.

In order to compare the effects of Se supply by foliar application, seedlings of the same size were transplanted into uniform (15-cm diam., 15-cm high) flowerpots filled with equivalent aforementioned soils (one seedling in each pot). The pots were divided into four groups (nine pots for each group) and the plants were supplied with 0, 50, 100, or 200 mg L^−1^ of Se (IV) by foliar application 15 and 30 day after transplanting, respectively. The pots were placed under a transparent plastic shed with appropriate watering. After transplanting for 45 day, the plants in each treatment were harvested for subsequent measurement.

### Sample preparation and biomass measurement

The roots and leaves of the treated plants were harvested, and the roots were washed with distilled water. The fresh weights of the root and leaf samples were measured, and the samples dried in an 80°C oven for 48 h prior to dry biomass measurement. A conversion factor for converting fresh weight of the experimental plants to dry weight was calculated. Three biological replications were performed for each sample.

### Se concentration determination

The total Se concentration in the leaves and roots for all treatment samples (excluding control samples) was determined by hydride generation-atomic fluorescence spectrometry (HG-AFS) based on the national food safety standard of China (GB 5009.93–2010). Briefly, approximately 0.5–1.0 g dried samples were added to digestion bottles. A 10-mL acid mixture (9:1 nitric acid: perchloric acid) was subsequently injected into the bottle and the samples were left to digest overnight. Afterwards, the mixtures were heated until the solutions ran clear (2 mL solution left). When the temperature cooled, the solutions were combined with 5.0 mL HCl (4.10) and subjected to continued heating until the solutions ran clear with emission of white smoke from the samples. When Se (VI) was reduced to Se (IV), the solutions were cooled and then transferred to 50-mL volumetric flasks and fixed to a volume of 50 mL after rinsing three times using ultrapure water. The blank control was treated using the same method. A 10-mL digestion solution was mixed with 2.0 mL HCl (4.30) and 1.0 mL ferricyanide solution (4.70) in a 15-mL centrifuge tube for determining concentration using an atomic fluorescence spectrometer. The Se concentrations were calculated according to a standard curve. To draw the standard curve, 0, 0.1, 0.2, 0.3, 0.4, and 0.5 mL of standard solutions (1 μg mL^−1^) were injected into 15-mL centrifuge tubes and volume fixed to 10 mL in each tube. The solutions were individually mixed with 2.0 mL HCl (4.30) and 1.0 mL ferricyanide solution (4.70) for detection. A standard curve was not drawn until the linear correlation coefficient was greater than 0.99. The detection limit of total Se was 0.02 mg kg^−1^. Three biological replications were performed for each sample.

### Parameter calculation

1. Bioconcentration factor (BCF) = Se concentration in plant (mg kg^−1^)/Se concentration in soil (mg kg^−1^) (Liao et al., 2013).
2. Translocation coefficient (TC) = Se concentration in leaf (mg kg^−1^)/Se concentration in root (mg kg^−1^) (Huang et al., 2012).
3. Daily intake of Se (μg) = *C*_Se_ × *C*_factor_ × *D*_foodintake_ × 1000. *C*_Se_, *C*_factor_, and *D*_foodintak_ represent the Se concentrations in plants (mg kg^−1^ DW), conversion factor, and daily intake of vegetables, respectively. The conversion factor 0.107 is used to convert fresh weight to dry weight for turnip fleshy roots in the present study. The average daily vegetable intake for adults was considered to be 0.345 kg person^−1^ day^−1^ (Parveen et al., 2015).
4. Optimum or maximum *C*_Se_ in turnip (mg kg^−1^ DW) = Optimum or maximum daily Se intake*/*(*C*_factor_ × *D*_foodintake_ × 1000). The optimum and maximum daily Se intake is 55–200 μg and 400 μg, respectively. *C*_Se_, *C*_factor_, and *D*_foodintak_ represent Se concentrations, conversion factor and daily intake of vegetables, respectively. The conversion factor 0.107 is used to convert fresh weight to dry weight for fleshy turnip roots in the present study. The average daily vegetable intake for adults is 0.345 kg person^−1^ day^−1^ (Parveen et al., 2015).

### Statistical analysis

Statistical analyses were performed using SPSS version 18.0. Parametric (one-way ANOVA or independent-samples *t*-test) or nonparametric (K independent-samples or 2 independent-samples) statistical tests, respectively, were applied.

The data were fitted to linear [y = ax+b] and polynomial [y = ax^2^+bx+c] models to analyze the correlations. Based on *R*^2^ and *P* values, the model (either linear or polynomial) best fit to the data was selected. All analyses were conducted with SigmaPlot 10.0.

## Results

### Effects of different treatment modes on turnip growth

**Figure 1:**
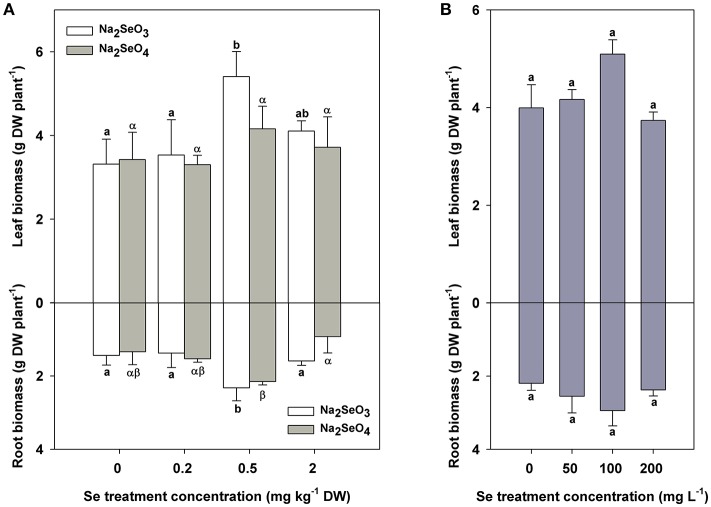
Biomass accumulation (g plant^−1^ DW) of turnip plants under different Se treatment modes. **(A)** Biomass differences of turnip leaves (top) and roots (bottom) treated with different concentrations of Se (IV) or Se (VI) by soil addition. **(B)** Biomass differences of turnip leaves (top) and roots (bottom) treated with different concentrations of Se (IV) by foliar application. Data represent means ± SE (*n* = 3). Bars labeled with different letters (a, b or α, β) are significantly different among different Se treatment concentrations of Se (IV) or Se (VI) (*P* < 0.05) **(A,B)**.

Leaf biomass was significantly greater when 0.5 mg kg^−1^ DW Se (IV) was added to soils (*P* < 0.05) whereas Se (VI) showed no significant effects on the leaf biomass accumulation in turnip at 0.2–2 mg kg^−1^ DW concentrations (Figure 1A top). The root biomass was markedly improved by both Se (IV) and Se (VI) at a concentration of 0.5 mg kg^−1^ DW in soil (*P* < 0.05) (Figure 1A bottom). However, the growth of turnips (both the leaves and roots) was not influenced by 50–200 mg L^−1^ Se (IV) used for foliar application in the present study (Figure 1B).

### Accumulation characteristics of Se (IV) and Se (VI) in turnip

**Figure 2:**
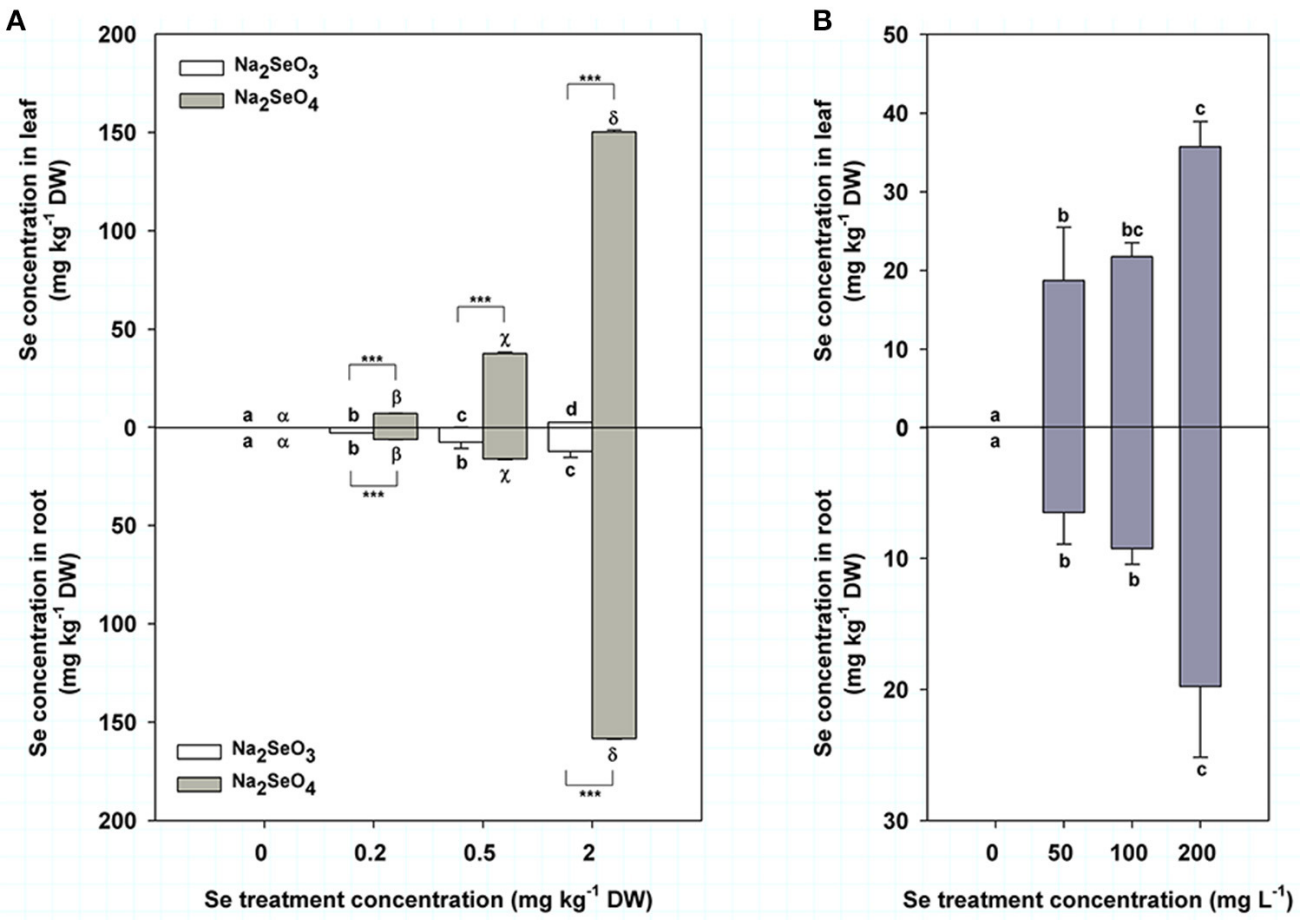
Se concentrations (mg kg^−1^ DW) in turnip plants in different Se treatment modes. **(A)** Se concentrations in turnip leaves (top) and roots (bottom) treated with different concentrations of Se (IV) or Se (VI) by soil addition. **(B)** Se concentrations in turnip leaves (top) and roots (bottom) treated with different concentrations of Se (IV) by foliar application. Data represent means ± SE (*n* = 3). Bars labeled with different letters (a–d or α-δ) are significantly different among different Se concentrations of Se (IV) or Se (VI) (*P* < 0.05) **(A,B)**. ^***^Represents a significant difference between two treatments (*P* < 0.001) **(A)**.

Se concentration in turnip leaves was 0.06, 0.27, and 2.73 mg kg^−1^ DW when treated by 0.2, 0.5, and 2 mg kg^−1^ DW Se (IV), respectively (Figure 2A top), while the values were 7.13, 37.53, and 150.33 mg kg^−1^ DW, respectively, under the same Se (IV) concentrations (Figure 2A top). Se concentration in turnip leaves was significantly increased with the increasing soil Se concentrations of both Se (IV) and Se (VI) (*P* < 0.05) (Figure 2A top). Additionally, we found that turnip leaves treated by Se (VI) accumulated obviously more Se compared with those treated by Se (IV) under the same concentration (*P* < 0.001) (Figure 2A top). Se concentrations in turnip roots showed similar change patterns with those in leaves (Figure 2A bottom), which ranged from 2.96 to 12.44 mg kg^−1^ DW and from 6.30 to 158.33 mg kg^−1^ DW in roots treated by Se (IV) and Se (VI), respectively (Figure 2A bottom). When treated by a foliar application of 50–200 mg L^−1^ Se (IV), turnip leaves and roots had Se concentrations of 18.71–35.70 mg kg^−1^ DW and 6.50–19.77 mg kg^−1^ DW, respectively (Figure 2B). Se concentrations were similar between 50 and 100 mg L^−1^ Se (IV) treatment but were significantly higher when the treatment concentration reached 200 mg L^−1^ (*P* < 0.05) (Figure 2B). Interestingly, the Se concentrations in both leaves and roots of turnips had highly significant positive correlations with the Se treatment concentrations in different modes (*P* < 0.01), which showed representative linear relations (Table 2).

**Table 2:** Results of regression analysis between exogenous Se treatment concentrations and Se accumulation concentrations in turnip leaves and roots.

**Treatment mode**	**Se form**	**Tissue**	**Regression equation**	***R*^2^**	**F**	***P***
Soil addition	Se (IV)	Leaf	*y* = 1.433x − 0.20	0.980	477.155	<0.001
		Root	*y* = 5.569xx + 1.998	0.594	14.654	0.003
	Se (VI)	Leaf	*y* = 76.691x − 3.016	0.997	3335.329	<0.001
		Root	*y* = 82.665x − 10.607	0.980	497.228	<0.001
Foliar application	Se (IV)	Leaf	*y* = 0.164x + 4.698	0.856	59.413	<0.001
		Root	*y* = 0.096x + 0.496	0.883	75.217	<0.001

**Figure 3:**
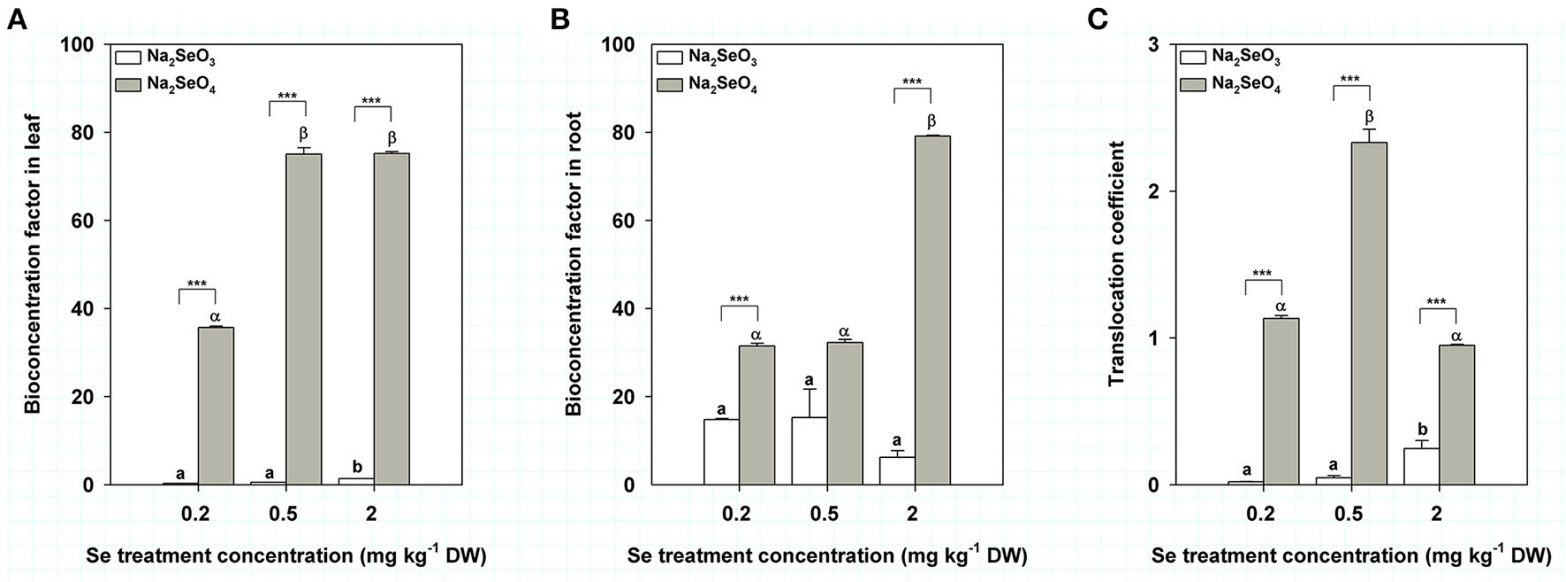
Se bioconcentration factors and translocation coefficients in turnip plants treated with different concentrations of Se (IV) or Se (VI) by soil addition. **(A)** Se bioconcentration factors in turnip leaves. **(B)** Se bioconcentration factors in turnip root. **(C)** Se translocation coefficients in turnip plants. Data represent means ± SE (*n* = 3). Bars labeled with different letters (a, b or α, β) are significantly different among different Se concentrations of Se (IV) or Se (VI) (*P* < 0.05) **(A–C)**. ^***^Represents a significant difference between two treatments (*P* < 0.001) **(A–C)**.

In order to reflect the ability of turnips to accumulate different Se forms, we calculated the BCFs in leaves and roots for Se (IV) and Se (VI) treatment by soil addition. The BCFs in leaves of turnip treated by Se (IV) (from 0.2 to 2 mg kg^−1^ DW) ranged from 0.31 to 1.36 whereas those of turnip treated with Se (VI) increased from 35.67 to 75.17 (Figure 3A). Under the same Se concentration, BCF of Se (VI) in turnip leaves was significantly higher than that of Se (IV) (*P* < 0.001) (Figure 3A). Interestingly, BCFs of Se (IV) in turnip roots were relatively stable but showed a decreasing trend (from 14.82 to 6.22) with increasing Se concentrations (from 0.2 to 2 mg kg^−1^ DW) (Figure 3B). However, the BCFs in roots of turnip treated with Se (VI) were similar between 0.2 and 0.5 mg kg^−1^ DW concentrations (31.50 and 32.27) but were significantly higher when the treatment concentration reached 2 mg kg^−1^ DW (79.17) (*P* < 0.05) (Figure 3B). As a result, the BCFs in turnip roots treated by Se (VI) were significantly higher than those of Se (IV) treatment samples under 0.2 and 2 mg kg^−1^ DW treatment concentrations (Figure 3B). TCs in turnips treated by Se (IV) were similar under 0.2 and 0.5 mg kg^−1^ DW concentrations (0.02 and 0.05) but were significantly higher when the treatment concentration reached 2 mg kg^−1^ DW (0.25) (Figure 3C); however, TCs in turnips treated by Se (VI) reached the maximum at 0.5 mg kg^−1^ DW concentration (2.33) while the values of 0.2 and 2 mg kg^−1^ DW concentrations were similar (1.13 and 0.95) (Figure 3C).

### Se biofortification potentiality analysis

Estimated daily Se intake levels are showed in Table 3. For adults, the daily intake of Se from turnip roots treated with 0.2–2 mg kg^−1^ DW Se (IV) added in soil was estimated to be 108.93–457.27 μg, while those from samples treated with Se (VI) was 231.58–5,820.05 μg (Table 3). The daily intake of Se from turnip roots treated with a foliar application of 50–200 mg L^−1^ Se (IV) ranged from 238.86 to 726.71 μg (Table 3).

**Table 3:** Daily intake of Se (μg) for adults from fleshy turnip roots.

**Treatment mode**	**Se form**	**Concentration**	**Daily intake of Se (μg)**
Soil addition	Se (IV)	0.2 mg kg^−1^ DW	108.93
		0.5 mg kg^−1^ DW	280.22
		2.0 mg kg^−1^ DW	457.27
	Se (VI)	0.2 mg kg^−1^ DW	231.58
		0.5 mg kg^−1^ DW	593.03
		2.0 mg kg^−1^ DW	5820.05
Foliar application	Se (IV)	50 mg kg^−1^ DW	238.86
		100 mg kg^−1^ DW	340.12
		200 mg kg^−1^ DW	726.71

*The daily intake of turnip root for adults used in the present study is 0.345 kg fresh weight*.

We also estimated optimum and maximum Se concentrations in turnip roots based on the optimum (55–200 μg) and maximum (400 μg) daily intake of Se for adults, and further estimated the corresponding exogenous Se treatment concentrations by different modes based on above analyzed linear equations (Table 2) between Se treatment concentrations and Se concentrations in plants. The optimum Se concentration in turnip roots was 1.50–5.44 mg kg^−1^ DW while the optimum Se concentration in turnip roots was 10.88 mg kg^−1^ DW (Table 4). Maximum Se treatment concentrations were 1.60 mg kg^−1^ DW, 0.26 mg kg^−1^ DW, and 108.42 mg L^−1^ for soil Se (IV), soil Se (VI), and foliar Se (IV) treatments, respectively (Table 4). Optimum Se treatment concentrations were 0.15–0.19 mg kg^−1^ DW and 10.44–51.62 mg L^−1^ for soil Se (VI) and foliar Se (IV) treatments, respectively (Table 4). Unfortunately, the values of optimum Se treatment concentrations for soil Se (IV) treatment were unavailable using the linear models (Table 4).

**Table 4:** Estimated optimum and maximum Se treatment concentrations for different treatment modes based on the optimum (55–200 μg) and maximum (400 μg) daily Se intake for adults from fleshy turnip roots (0.345 kg fresh weight).

**Optimum Se concentration in plant (mg kg^−1^ DW)**	**Optimum Se treatment concentration**	**Maximum Se concentration in plant (mg kg^−1^ DW)**	**Maximum Se treatment concentration**	**Treatment mode**
1.50–5.44	Unavailable	10.88	1.60 mg kg^−1^ DW	Se (IV) (Soil)
	0.15–0.19 mg kg^−1^ DW		0.26 mg kg^−1^ DW	Se (VI) (Soil)
	10.44–51.62 mg L^−1^		108.42 mg L^−1^	Se (IV) (Foliar)

*The treatment methods and harvest time were assumed to be those used in the present study*.

## Discussion

### Effects of Se on turnip growth

Although Se is not an essential element for angiosperms, it is considered to be a beneficial element since it can stimulate growth, confer tolerance to abiotic stresses, and provide resistance to pathogens or herbivory (Quinn et al., 2007; Pilon-Smits et al., 2009; Feng et al., 2013). Some studies have reported that Se could improve plant growth and grain production (Lyons et al., 2009). However, Se toxicity also has been often observed (Ximenez-Embun et al., 2004; Fu et al., 2011; Mao et al., 2011; Longchamp et al., 2015). For example, when grown with 12 μmol L^−1^ selenite, white lupine and sunflower were reported to experience a biomass reduction of 20% and 40%, respectively (Ximenez-Embun et al., 2004). Indeed, the effects of Se on plant growth usually show the principle of low-concentration promotion and high-concentration inhibition; this has been observed in many plants including radish, Chinese cabbage, pakchoi, rapeseed, and spinach (Fu et al., 2011; Mao et al., 2011). The results of our study supported this assertion, where both selenite and selenate had optimal promotion effects at 0.5 mg Se kg^−1^ dry soil and then began to inhibit plant growth as the Se concentrations increased. The results of Se (IV) treatment by foliar application also indicated that there was an optimum concentration for turnip growth near 100 mg L^−1^. Dhillon and Dhillon (2009) obtained similar results, in which the authors found that dry matter accumulation of turnip and several other vegetables decreased when 1.25–5.0 mg kg^−1^ selenate-Se was applied to the soil (Dhillon and Dhillon, 2009). The effect intensity of Se (IV) vs. Se (VI) on plant growth is inconclusive. Mao et al. (2011) found that Se (VI) had a significant promoting effect on cauliflower at low concentrations and lower toxicity to wheat and alfalfa at high concentrations compared with Se (IV) (Mao et al., 2011). Longchamp et al. (2015) also reported that Se (VI) produced lower inhibition effect on the growth of maize crops compared to Se (IV) at 12 μM Se concentration (Longchamp et al., 2015). However, we found similar effects of Se (IV) vs. Se (VI) on turnip growth at 0.2–2 mg kg^−1^ Se concentrations. This might be because the range of Se concentrations for promoting or inhibiting plant growth was greatly distinct in different species, attributed to the differences of plant tolerance to Se or various environmental conditions.

### Se absorption and translocation in turnip

Se (IV) and Se (VI) are two main Se forms in the natural soils and can be absorbed by plant roots (White and Broadley, 2009). Se forms and soil environment, especially soil pH and pe, greatly affect Se uptake and accumulation in plants (Longchamp et al., 2015). Our results showed that turnips more easily accumulated Se (VI) in the soil humus in comparison with Se (IV). This was consistent with previous reports finding that Se (VI) was relatively mobile in soil solution and thus the addition of Se (VI) to soils facilitated immediate Se accumulation by plants while Se (IV) provided a longer-lasting Se source (Broadley et al., 2006). However, many studies on rice, wheat, soybean, or maize have also shown that Se (IV) can accumulate as much as (Zayed et al., 1998; Li et al., 2008) or even more than (Zhang et al., 2003; Longchamp et al., 2015) Se (VI). For example, Longchamp et al. (2015) found that maize absorbed more Se (IV) than Se (VI) in whole plants, as well as in roots and grains, although the Se concentrations in stems and leaves supplied with Se (VI) were much higher than those supplied with Se (IV) (Longchamp et al., 2015). Our results also agreed with findings that Se contents in plants were dependent on the Se concentrations in soils (Fu et al., 2011; Schiavon et al., 2016). In the present study, the Se concentration in roots and leaves of turnip plants was significantly positively correlated with Se (IV) or Se (VI) treatment concentrations applied either by soil addition or foliar application. Plant accumulation ability is essential for both biofortification and phytoremediation (Wu et al., 2015). To date, only a few studies have definitely provided data about Se accumulation in turnips worldwide. An American study reported that Se concentrations in turnips were 60 mg kg^−1^ DW in the presence of 2 mg L^−1^ Se in irrigation water and Se concentrations in turnip shoots irrigated by sprinkler were about twice the amount in flood-irrigated plants(Suarez et al., 2003). Unfortunately, these results are different compared to our findings that Chinese turnips accumulated relatively low Se concentrations (about 20–30 mg kg^−1^ DW) irrigated by sprinkler using as high as 200 mg L^−1^ Se (IV). This might be attributed to complex reasons, including diverse accumulation abilities in turnip genotypes and the differences in solution compositions. In an Indian study, scientists reported that turnip accumulated 60–70 mg kg^−1^ Se both in edible and inedible portions when the soil was fertilized with 2.5 mg kg^−1^ selenate-Se (Dhillon and Dhillon, 2009). The results were also inconsistent with our findings that Chinese turnips accumulated less than 15 mg kg^−1^ DW Se (IV) but more than 150 mg kg^−1^ DW Se (VI) in both roots and leaves at the similar Se concentrations added to the soil. However, Liu et al. (2012) found that turnips from the Tibet region contained 6.33 mg kg^−1^ Se (Liu et al., 2012); this is an interestingly high value for local areas lacking soil Se. Although significant differences have been observed in different reports worldwide, these results have indicated that turnip has an ability to accumulate Se under artificial or natural conditions. We found that turnip could tolerate more than 150 mg kg^−1^ DW Se in both leaves and roots, which did not significantly affect the plant growth. The results indicate that turnip might belong to potential Se-indicator species as defined in a previous report (White, 2016).

BCF is usually used to reflect the ability of plants to absorb trace elements from the soil environment (Liao et al., 2013). Our results showed that the maximum BCFs of Se (IV) and Se (VI) in turnip roots reached 15 and 79, respectively, reflecting an ability of turnips to absorb Se, but a stronger ability for Se (VI). TC is used to reflect transport of elements or ions in plants (He, 2013), which is closely related to the intake risks of contaminants in leaf vegetables, as well as phytoremediation efficiency for polluted environments (Gao et al., 2012). Interestingly, the TCs of Se (IV) were far lower than 1 whereas those of Se (VI) were about 1–2. These results indicated a differential ability to transport different forms of Se in turnips. This might be attributed to a differential metabolism and transformation of Se (IV) and Se (VI) in turnips (Wu et al., 2015). In plants, Se (IV) is thought to be rapidly converted to organoselenium compounds in the root whereas Se (VI) is delivered immediately to the xylem (White et al., 2004; Ximenez-Embun et al., 2004; Li et al., 2008) and subsequently assimilated into organoselenium compounds in plastids (Pilon-Smits and Leduc, 2009). With Se (IV) treatment, organoselenium compounds might therefore be produced to a greater extent vs. production in the Se (VI) treatment. Longchamp et al. (2015) found no trace of inorganic Se could be detected in whole plants treated by Se (IV) (Longchamp et al., 2015). However, when treated with Se (VI), the percentage of Se (VI) in roots, stems and leaves was respectively 20, 54, and 39% (Longchamp et al., 2015). In several studies, traces of Se (IV) were only detected at less than 7%, indicating that Se (IV) in plants overwhelmingly consists of organoselenium compounds (Ximenez-Embun et al., 2004). However, some studies have also shown that about 30–97% of Se in the leaves, stems or roots consists of Se (IV) (Ximenez-Embun et al., 2004; Li et al., 2008; Mazej et al., 2008; Longchamp et al., 2015). Thus, further analysis of the metabolism and transformation processes of different Se forms in turnips is needed. More interestingly, we found that the BCFs of Se (IV) in leaves, as well as those of Se (VI) in both leaves and roots, showed maximum values at the highest Se treatment concentration (2.0 mg kg^−1^ DW) used in the present study. A similar result was also observed for the TC value of Se (IV), whereas that of Se (VI) reached the maximum at 0.5 mg kg^−1^ DW Se treatment concentration. These results indicate that higher Se content in soils further improved bioavailability of Se, but this requires further verification and exploration.

### Se biofortification potentiality in turnip

Producing and consuming the biofortified agricultural products has been proposed as a promising functional agricultural strategy to increase dietary nutrient intake, e.g., Se, for humans (Wu et al., 2015). Biofortification of Se is closely connected to enhancing the efficiency of Se uptake and accumulation in plants (Vamerali et al., 2014). The Tibetan Plateau is famous for having a hard-living environment for higher organisms, including animals and humans. Unfortunately, most parts of the plateau are considered to be areas where Se deficiency disease frequently occurs (Zhu et al., 2009). Turnip is a cruciferous vegetable mainly distributed around the Tibetan Plateau in China; through biofortification it may be an excellent candidate to supplement daily Se requirement for local people, since cruciferous plants usually have strong abilities to accumulate micronutrients (e.g., Se, zinc, and iron; White and Broadley, 2009). The results of the present study and several previous reports indicate that turnip has a relatively strong ability to accumulate Se (Dhillon and Dhillon, 2009; Liu et al., 2012) and show that turnip is a possible candidate for developing Se-functional products. In addition, turnip is rich in nutrients like vitamins, mineral elements, and amino acids (Liu et al., 2012; Ma et al., 2016) and contains a variety of medicinal ingredients (Li et al., 2011; Hu et al., 2016; Ma et al., 2016). Moreover, Se has been found to have beneficial effects in promoting plant nutrition and metabolites (Zhao et al., 2003, 2004). For example, Se can improve the synthesis of glucosinolates, important secondary metabolites found mainly in cruciferous plants, by replacing the sulfur element and promoting the activities of some enzymes (Robbins et al., 2005; Barickman et al., 2013; Avila et al., 2014). Se fertilizer concentrations that are beneficial for turnip are therefore greatly recommended. Moreover, Se also has been demonstrated to improve the tolerance of plants to common abiotic stresses including drought, cold and heavy metals (Chu et al., 2010; Al-Waeli et al., 2012; Lin et al., 2012; Ahmad et al., 2016). This is also worth considering for turnip growth and yield in the Tibetan Plateau.

According to the recommended optimum dietary allowance of Se (55–200 μg Se d^−1^) and the maximum critical safety value (400 μg Se d^−1^) for adults (Wu et al., 2015), we estimated the daily Se intake for adults based on the Se concentrations in the turnip roots in the present study, which were the main edible portions for local people. Unfortunately, the results produced by most samples exceeded thresholds for supplying daily Se intake. Comparatively, the samples treated by Se (VI) would be much more likely to cause selenosis vs. those treated by Se (IV), either with soil addition or foliar spraying. Thus, Se (IV) should be a priority selection as artificial Se fertilizer for turnip under the similar soil conditions. As Se concentrations in turnip roots were significantly positively correlated with the exogenous Se treatment concentrations, we also estimated the optimum and maximum Se concentrations for treating turnips used in adult diets based on the linear regression equations. The results indicated that the safe Se concentrations for Se (IV) and Se (VI) treated by soil addition were below 1.60 and 0.26 mg kg^−1^ DW, respectively, whereas that for Se (IV) treated by foliar application should be lower than 108.42 mg L^−1^. The results provide some useful information for Se biofortification in turnip. However, as mentioned above, organic Se is the most healthy Se form for humans; transformation efficiency of the inorganic Se (IV) and Se (VI) in turnips is unknown. Furthermore, soil properties have been demonstrated to affect Se accumulation and transformation (Vamerali et al., 2014; Wu et al., 2015). In addition, stage of plant growth at treatment, as well as treatment frequency and the duration of post-treatment growth periods, is also closely related to the accumulation of Se in plants. Thus, much more effort is needed to determine the characteristics of turnips to Se and accurately evaluate their practicability for producing Se-enriched foodstuff. However, our results have provided preliminary information and narrowed the scope of relevant research.

## Conclusions

In the present study, we compared the absorption and translocation differences of selenite and selenate in turnip. The results showed that the Se contents in both leaves and roots of turnip were significantly positively correlated with Se treatment concentrations either by soil addition or by foliar spraying. The biomass of turnips was improved at a concentration of 0.5 mg Se (IV) or Se (VI) kg^−1^ dry soil. Moreover, turnip absorbed markedly more Se (VI) than Se (IV) in the same soils and much more Se (VI) could transfer from roots to leaves in comparison to Se (IV). Based on the relatively high Se concentrations, we considered that turnip might be a potential Se indicator plant, but supporting data is still needed from natural conditions. Results of BCFs and TCs also indicated that higher Se content in soils seemed to further improve bioavailability of Se. According to the optimum and maximum dietary allowance of Se for adults, we estimated the daily Se intake for adults based on our results. Although the Se concentrations in most root samples in this study were too high to be ingested by humans, we consider that Se (IV) should be a priority selection as an artificial Se fertilizer for turnips. In addition, the optimum and maximum Se concentrations for treating turnips were also estimated based on the linear relations between Se concentrations in turnip roots and Se treatment concentrations. The results provided some useful information for Se intake from turnips, but more effort is required to assess the practicability of using fortified turnips for producing Se-enriched foodstuff; open questions include a lack of transform efficiency of inorganic Se (IV) and Se (VI), the effects of soil properties on Se accumulation and transformation, and the effects of treatment stage of plant growth, treatment lasting time and times. Regardless, our results provide preliminary information and narrow the scope of relevant research.

## Author contributions

YPY and XL conceived and designed the experiments. XL, YW, and BL performed the experiments. XL analyzed the data. XL wrote the manuscript. YHY supported the data determination.

### Conflict of interest statement

The authors declare that the research was conducted in the absence of any commercial or financial relationships that could be construed as a potential conflict of interest.

## Abbreviations

BCF: bioconcentration factor
DW: dry weight
Se: selenium
TC: translocation coefficient.
